# A machine learning-based predictive nomogram for early neurological improvement after thrombolysis in acute ischemic stroke

**DOI:** 10.3389/fneur.2025.1662498

**Published:** 2025-11-12

**Authors:** Bing-Hua Lv, Hao-wei Deng, Zuo-yv Qin, Ning-qin Meng, Gui-ming Weng, Rui-Ting Hu, Chao Qin

**Affiliations:** 1 Department of Neurology, The First Affiliated Hospital of Guangxi Medical University, Nanning, China; 2 Department of Rheumatology and Immunology, The First Affiliated Hospital of Guangxi Medical University, Nanning, China; 3 Department of Neurology, Minzu Hospital of Guangxi Medical University, Nanning, China

**Keywords:** early neurological improvement, predictive model, machine learning algorithms, acute ischemic stroke, intravenous thrombolysis

## Abstract

**Background:**

Early neurological improvement (ENI) is a critical prognostic indicator for acute ischemic stroke (AIS) patients undergoing intravenous thrombolysis with recombinant tissue plasminogen activator (rt-PA). This study aimed to develop and validate a machine learning (ML)-based model for predicting ENI using clinical and biochemical data.

**Methods:**

Clinical data from 217 AIS patients (97 ENI, 120 non-ENI) were retrospectively analyzed. Significant baseline differences were identified between groups, including hemorrhage, onset-to-needle time (ONT), neutrophil-to-lymphocyte ratio (NLR), weight, and activated partial thromboplastin time (APTT). Four ML algorithms, including Multilayer Perceptron (MLP), Random Forest (RF), Support Vector Machine (SVM), and XGBoost, were implemented. Model performance was evaluated via area under the receiver operating characteristic curve (AUC). Key predictors were identified by intersecting top-ranked features from all algorithms, followed by logistic regression modeling and nomogram visualization.

**Results:**

The MLP model achieved the highest AUC (0.77) in the testing set, outperforming RF (0.72), SVM (0.63), and XGBoost (0.68). Six overlapping parameters, including APTT, ALT/AST ratio, ONT, mean corpuscular hemoglobin concentration (MCHC), weight, and NLR, were selected as core predictors. The logistic regression model incorporating these parameters yielded an AUC of 0.74, while the nomogram demonstrated that the predictive model exhibited strong discriminative ability (C-index: 0.817) for predicting ENI in rt-PA-treated AIS patients.

**Conclusion:**

This ML-based model effectively predicts ENI in rt-PA-treated AIS patients by integrating critical clinical and biochemical markers. Its application may optimize personalized treatment strategies, enhance clinical decision-making, and improve patient outcomes.

## Introduction

In recent years, the administration of recombinant tissue plasminogen activator (rt-PA) for intravenous thrombolysis has become a cornerstone in the acute management of ischemic stroke (1). Numerous studies have highlighted the importance of early neurological improvement (ENI) as a predictor of long-term outcomes and functional independence (2, 3). Research has shown that patients who exhibit rapid improvement in their neurological status within the first few hours post-treatment are more likely to achieve favorable outcomes (4). The mechanisms underlying ENI are complex and multifactorial, involving reperfusion of ischemic but viable brain tissue, reduction of infarct size, and preservation of the blood–brain barrier (5, 6). Moreover, several blood biomarkers are being explored to identify patients most likely to benefit from rt-PA and to predict early response to treatment.

Machine learning (ML) algorithms have emerged as powerful tools in the construction of predictive models for adverse events following acute ischemic stroke (AIS), offering significant advantages over traditional statistical approaches (7, 8). By leveraging complex, non-linear relationships within large and diverse datasets, ML algorithms can identify subtle patterns and risk factors that may not be apparent through conventional analysis, thereby enhancing the accuracy and robustness of prediction. Techniques such as logistic regression, decision trees (DT), random forests (RF), support vector machines (SVM), and neural networks have been applied to forecast complications like hemorrhagic transformation, recurrent stroke, and mortality (9–11). The use of ML also supports real-time predictions and personalized medicine, enabling timely interventions to mitigate adverse outcomes.

To date, several studies reported machine-learning based prediction of future outcome in stroke patients. For instance, Wen et al. (12) reported that the model constructed by two machine-learning served as robust tools for predicting early neurological deterioration in acute ischemic stroke patients following thrombolysis. Moreover, Fan et al. (13) used four ML methods to screen and recombine the features for construction of prognostic model, and found that this model offers improved prediction accuracy that may reduce rates of misdiagnosis and missed diagnosis in patients with AIS. Regarding ENI in AIS patients undergoing rt-PA treatment, although three studies (14–16) revealed that several clinical indexes, such as diabetes mellitus history, kynurenic acid and kynurenine aminotransferase were associated with the ENI, no studies using ML algorithms to construct predictive model for ENI in AIS patients undergoing rt-PA treatment. In addition, these previous models largely rely on a limited number of variables and traditional statistical methods (e.g., logistic regression), which may not adequately capture the complex, non-linear relationships among multiple prognostic factors. Therefore, our study addresses this gap by incorporating a comprehensive set of clinical, laboratory variables and applying multiple ML algorithms to better model these interactions, thereby enhancing predictive accuracy.

## Methods

### Patient selection

**Figure 1:**
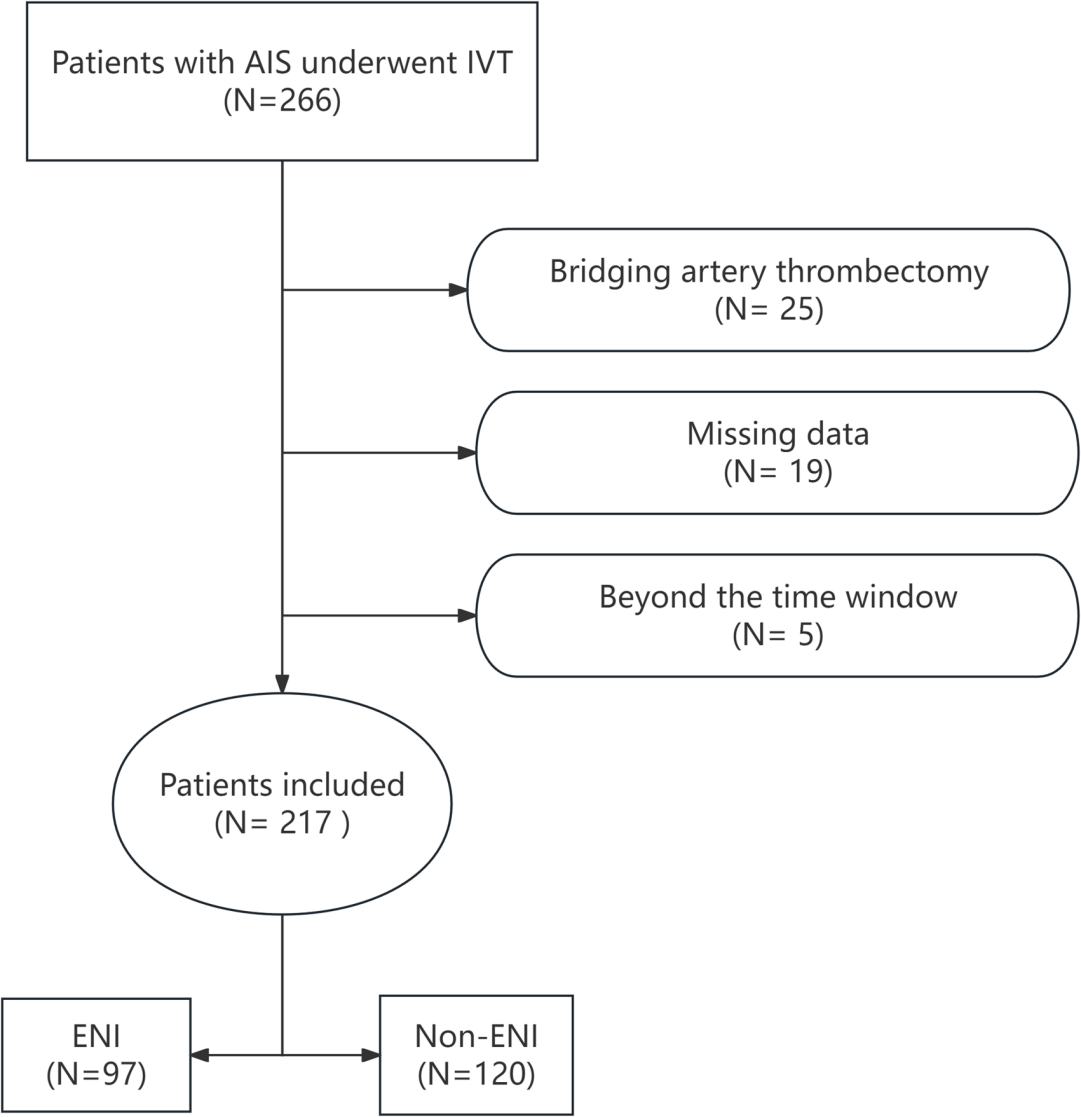
Flow chart of the study population.

The study adhered to the Declaration of Helsinki and was approved by the Ethics Committee of our hospital. A retrospective analysis was conducted on 266 patients with AIS who underwent rt-PA intravenous thrombolysis (IVT) at our hospital’s Stroke Center between June 1, 2020, and November 30, 2024. Inclusion criteria were as follows: individuals aged ≥18 years with a confirmed diagnosis of AIS based on CT or MRI, who received IVT within 4.5 h of stroke onset. Patients were excluded if they received bridging endovascular treatment after IVT, had incomplete clinical or laboratory data, or were discharged or deceased within 24 h. After excluding 25 patients who underwent bridging artery thrombectomy, 19 with incomplete clinical data, and 5 who were beyond the 4.5-h thrombolysis time window, a total of 217 patients who received rt-PA IVT were included in the study. The flow chart for patient selection is shown in Figure 1.

### Collection of clinical data and definition of ENI

Clinical data were collected for each patient, including demographics (age, gender, height, weight, and BMI), comorbidities, the rt-PA Dosage (0.9 or 0.6 mg/kg), baseline NIHSS score, medication history, and initial laboratory tests (blood routine examination, biochemical examination, liver function test, coagulation test, renal function test, electrolyte test, and lipid test). We also calculated inflammatory cell ratios: NLR (neutrophil-to-lymphocyte ratio), PLR (platelet-to-lymphocyte ratio), and LMR (lymphocyte-to-monocyte ratio). ENI was defined as an NIHSS score decrease of ≥4 points within 24 h of hospitalization or complete recovery within 24 h (17).

### Variable selection and establishment of machine learning models

Study participants were randomly divided into training (80%) and testing (20%) sets. Clinical data were standardized, and four ML algorithms—Multilayer Perceptron (MLP), Random Forest (RF), Support Vector Machine (SVM), and XGBoost—were applied to construct predictive models for ENI and screen key parameters using the clinical data. A total of 68 clinical and laboratory variables were included in the initial ML screening phase, including both raw measurements and derived ratios (such as NLR, PLR, ALT/AST). Although some variables were biologically or mathematically related, they were retained in the initial screening to avoid premature exclusion of potentially informative features. The performance of the models was quantitatively evaluated using receiver operating characteristic (ROC) curves, with the area under the curve (AUC) serving as the primary metric. The selected clinical indicators were then integrated into a logistic regression classification algorithm. A nomogram was generated to visualize the predictive value of each parameter.

For data preprocessing, the missing data were handled using multiple imputation by chained equations (MICE), and outliers were identified using the interquartile ranges (IQRs) method and winsorized where appropriate. Continuous variables were standardized using z-score normalization prior to model training. A grid search with 5-fold cross-validation was used to optimize key parameters for each ML algorithm. The optimal parameters were selected based on the highest cross-validated AUC value. The SHapley Additive exPlanations (SHAP) Python package (version 0.40.0) was used to measure the effects of the parameters on the predictive model, assessing feature importance using a game-theoretic approach.

To mitigate overfitting due to high dimensionality, we employed a conservative feature selection strategy by intersecting the top 20 features ranked by each of the four ML algorithms, resulting in a final set of 6 variables for logistic regression modeling.

### Statistical analysis

Statistical analyses were conducted using R software (version 4.2.2). Continuous variables were presented as mean ± standard deviation (SD) or IQRs, while categorical variables were expressed as percentages (n, %). Continuous variables were assessed using *t*-tests or non-parametric Mann–Whitney U tests, as appropriate, while chi-square tests were used for categorical variables to compare baseline characteristics between the ENI and non-ENI groups. To assess potential multicollinearity among the final set of six predictors, we computed the Variance Inflation Factor (VIF) for each variable in the logistic regression model. Significant differences were considered at *p* < 0.05.

## Results

### Comparison of clinical data between ENI and non-ENI patients

Table 1 summarizes the baseline characteristics of both groups. The study included 217 patients were divided into ENI group (*n* = 97) and non-ENI group (*n* = 120) based on the achievement of ENI. Baseline characteristics comparison revealed that ENI group patients were younger (mean age 62 vs. 67.5 years) with a higher proportion of males (74.2% vs. 66.7%). There was significantly shorter onset-to-treatment time in ENI group compared with non-ENI group (150 vs. 174 min). Lower value of NEU (5.12 vs. 5.62), NEU% (66% vs. 70%) and NLR (2.81 vs. 4.03). Higher LYM (0.23 vs. 0.18), LYM% (1.73 vs. 1.52), LMR (3.16 vs. 2.48), MCV (87.99 vs. 90.39), and Fasting GLU (5.17 vs. 5.71) were found in ENI group than in non-ENI group (*p* < 0.05). However, higher value of weight (67.5 vs. 62.5), BMI (24.51 vs. 23.25), APTT (30.9 vs. 29.7), A/G ratio (1.40 vs. 1.30), PA (256.89 vs. 232.67), and CHE (8623.64 vs. 8,043) were found in ENI group than in non-ENI group (*p* < 0.05). However, no significant differences were found in resting indexes (*p* > 0.05). These findings suggest that younger age, shorter thrombolysis time window, reduced inflammatory status, better nutritional/metabolic condition, and appropriate anticoagulation status may be closely associated with early neurological improvement following intravenous thrombolysis.

**Table 1:** Comparison of clinical data between ENI and non-ENI patients.

Variables	Total	ENI (*N* = 97)	Non-ENI (*N* = 120)	*P-*value
Demographics				
Age, years	64.0 ± 12.9	62.0 (54.0–71.0)	67.5 (58.0–75.0)	0.050
Gender				0.289
Female	65 (30.0%)	25 (25.8%)	40 (33.3%)	
Male	152 (70.0%)	72 (74.2%)	80 (66.7%)	
Physiological characteristics or status				
Height, cm	163.9 ± 10.5	167.0 (158.0–170.0)	165.0 (160.0–170.0)	0.224
Weight, kg	65.7 ± 14.1	67.50 (60.0–75.0)	62.50 (55.0–70.0)	0.017
BMI, kg/m^2^	25.7 ± 24.8	24.51 (22.38–26.78)	23.25 (21.12–25.87)	0.015
Smoking	104 (47.9%)	53 (54.6%)	51 (42.5%)	0.100
Drinking	90 (41.5%)	40 (41.2%)	50 (41.7%)	1.000
Pre systolic	151.7 ± 22.2	151.0 (138.0–164.0)	154.50 (136.0–167.0)	0.405
Pre diastolic	86.5 ± 13.3	86.82 ± 12.61	86.31 ± 13.88	0.777
Key parameters of IVT				
Baseline NIHSS	7.4 ± 5.0	7.0 (3.0–10.0)	6.0 (4.0–10.0)	0.483
NIHSS (24 h after IVT)	4.9 ± 5.0	2.0 (0.0–4.0)	5.0 (3.0–11.0)	<0.01
rt-PA dosage (0.6 mg/kg)	44 (20.3%)	20 (20.6%)	24 (20%)	1.000
ONT, min	168.1 ± 59.0	150.0 (113.0–193.0)	174.0 (126.50–217.0)	0.035
DNT, min	68.5 ± 52.3	52.0 (39.0–72.0)	56.0 (37.50–89.0)	0.301
Comorbidities				
Hypertension, n (%)	153 (70.5%)	62 (63.9%)	91 (75.8%)	0.078
Diabetes, n (%)	62 (28.6%)	23 (23.7%)	39 (32.5%)	0.203
Atrial Fibrillation, n (%)	21 (9.7%)	9 (9.3%)	12 (10%)	1.000
intracerebral hemorrhage after thrombolysis, n (%)	26 (12.0%)	6 (6.2%)	20 (16.7%)	0.031
Baseline laboratory test				
GLU, mmol/L	8.0 ± 3.3	7.06 (6.01–8.30)	7.0 (6.20–8.59)	0.809
WBC, 10^9^/L	8.7 ± 3.0	7.87 (6.77–9.29)	8.21 (6.89–9.84)	0.121
RBC, 10^12^/L	4.6 ± 0.7	4.65 (4.33–5.08)	4.46 (4.18–4.79)	0.070
HGB, g/L	130.8 ± 19.0	133.40 (121.0–146.0)	132.70 (117.50–144.0)	0.587
MCV, fl	87.2 ± 9.2	87.99 (83.61–91.66)	90.39 (86.20–93.92)	0.040
MCHC, g/L	28.8 ± 3.7	29.30 (26.77–30.58)	30.27 (28.12–31.56)	0.070
PLT, 10^9^/L	235.8 ± 72.4	241.0 (189.70–286.60)	222.40 (186.80–271.50)	0.206
NEU, 10^9^/L	6.1 ± 2.9	5.12 (3.91–6.44)	5.62 (4.48–7.71)	0.024
NEU%	0.7 ± 0.1	0.66 ± 0.11	0.70 ± 0.11	0.040
LYM, 10^9^/L	1.7 ± 0.7	1.73 (1.35–2.24)	1.52 (1.12–1.83)	0.010
LYM%	0.2 ± 0.1	0.23 (0.17–0.28)	0.18 (0.12–0.26)	0.010
MONO, 10^9^/L	0.6 ± 0.2	0.59 (0.47–0.75)	0.62 (0.50–0.81)	0.129
NLR	4.6 ± 3.7	2.81 (2.15–4.29)	4.03 (2.27–6.49)	0.020
PLR	161.5 ± 81.2	131.65 (95.06–183.45)	151.20 (116.18–204.36)	0.085
LMR	2.9 ± 1.4	3.16 (2.12–4.11)	2.48 (1.62–3.40)	<0.01
TBiL, μmol/L	12.7 ± 7.1	11.20 (7.90–14.40)	11.80 (8.20–15.95)	0.487
DBiL, μmol/L	3.8 ± 2.7	3.20 (2.40–4.10)	3.20 (2.30–4.80)	0.559
IBil, μmol/L	8.8 ± 5.3	7.60 (5.30–10.90)	8.20 (5.70–11.70)	0.459
DB/TB ratio	0.3 ± 0.1	0.30 (0.24–0.37)	0.29 (0.23–0.37)	0.841
TP, g/L	68.8 ± 6.0	69.26 ± 5.91	68.49 ± 5.99	0.348
ALB, g/L	38.6 ± 4.6	39.20 (36.80–42.10)	38.35 (34.90–41.15)	0.061
GLO, g/L	29.9 ± 4.4	29.10 (26.50–31.60)	29.50 (26.90–33.30)	0.189
A/G ratio	1.3 ± 0.3	1.40 (1.20–1.50)	1.30 (1.10–1.45)	0.020
GGT, U/L	39.6 ± 53.7	26.0 (20.0–37.0)	28.0 (16.50–44.50)	0.377
TBA, μmol/L	6.7 ± 10.7	4.10 (2.50–6.70)	3.85 (2.20–7.55)	0.905
AST, U/L	25.9 ± 19.5	20.0 (17.0–26.0)	23.0 (18.0–28.0)	0.105
ALT, U/L	23.6 ± 27.7	18.0 (13.0–23.0)	15.50 (12.0–26.0)	0.244
ALT/AST	1.4 ± 0.8	1.10 (0.90–1.40)	1.40 (0.90–1.90)	0.090
ALP, U/L	77.1 ± 24.3	74.0 (57.0–86.0)	74.75 (61.0–91.50)	0.312
PA, mg/L	243.5 ± 69.2	256.89 ± 65.95	232.67 ± 70.16	0.010
CHE, U/L	8302.9 ± 2025.7	8623.64 ± 1980.39	8043.66 ± 2032.98	0.036
CREA, μmol/L	87.2 ± 68.3	80.0 (65.0–93.0)	76.0 (65.50–96.0)	0.369
UA, μmol/L	329.7 ± 105.2	332.44 ± 97.90	327.44 ± 111.04	0.728
K, mmol/L	3.9 ± 0.4	3.85 (3.69–4.07)	3.84 (3.69–4.12)	0.900
Na, mmol/L	140.1 ± 4.2	140.0 (138.50–141.30)	140.0 (137.15–141.90)	0.912
CL, mmol/L	106.6 ± 4.7	106.50 (104.4–108.60)	106.15 (104.40–108.60)	0.612
Ca, mmol/L	2.2 ± 0.1	2.20 (2.12–2.28)	2.21 (2.13–2.28)	0.393
Mg, mmol/L	0.8 ± 0.1	0.83 (0.77–0.88)	0.82 (0.77–0.88)	0.742
P, mmol/L	1.0 ± 0.2	1.01 ± 0.24	0.99 ± 0.26	0.551
APTT, s	30.3 ± 3.9	30.90 (28.10–33.10)	29.70 (27.15–32.30)	0.049
PT, s	11.5 ± 1.0	11.20 (10.80–12.0)	11.50 (10.90–12.35)	0.070
FIB, g/L	3.5 ± 1.2	3.25 (2.67–3.83)	3.42 (2.78–4.30)	0.109
TT, s	13.2 ± 2.8	12.50 (11.50–13.70)	12.45 (11.45–14.30)	0.695
INR	1.0 ± 0.1	0.99 (0.94–1.05)	1.01 (0.94–1.08)	0.189
PTA, %	98.6 ± 14.7	100.32 ± 13.83	97.20 ± 15.30	0.121
T-CHO, mmol/L	4.8 ± 1.2	4.64 (4.01–5.26)	4.77 (4.05–5.51)	0.181
TG, mmol/L	1.6 ± 1.2	1.37 (1.0–1.86)	1.38 (1.06–1.86)	0.803
HDL-C, mmol/L	1.2 ± 0.3	1.13 (0.99–1.25)	1.18 (1.01–1.34)	0.107
LDL-C, mmol/L	2.9 ± 0.9	2.75 (2.25–3.52)	2.83 (2.38–3.40)	0.333
Fasting GLU, mmol/L	6.0 ± 2.3	5.17 (4.69–6.08)	5.71 (4.88–6.69)	0.017
TyG index	2.1 ± 0.6	2.05 (1.67–2.35)	2.08 (1.73–2.55)	0.166

NIHSS, National Institutes of Health Stroke Scale; BMI, Body Mass Index; ONT, Onset-to-Needle Time; DNT, door to needle time; GLU, Glucose; WBC, White Blood Cell; RBC, Red Blood Cell; HGB, Hemoglobin; MCV, Mean Corpuscular Volume; MCHC, Mean Corpuscular Hemoglobin Concentration; PLT, Platelet; NEU, Neutrophil; LYM, Lymphocyte; MONO, Monocyte; NLR, Neutrophil-to-Lymphocyte Ratio; PLR, Platelet-to-Lymphocyte Ratio; LMR, Lymphocyte-to-Monocyte Ratio; TBiL, Total Bilirubin; DBiL, Direct Bilirubin; IBil, Indirect Bilirubin; TP, Total Protein; ALB, Albumin; GLO, Globulin; GGT, Gamma-Glutamyltransferase; TBA, Total Bile Acids; AST, Aspartate Aminotransferase; ALT, Alanine Aminotransferase; ALP, Alkaline Phosphatase; PA, Prealbumin; CHE, Cholinesterase; CREA, Creatinine; UA, Uric Acid; APTT, Activated Partial Thromboplastin Time; PT, Prothrombin Time; FIB, Fibrinogen; TT, Thrombin Time; INR, International Normalized Ratio; PTA, Prothrombin Activity; T-CHO, Total Cholesterol; TG, Triglyceride; HDL-C, High Density Lipoprotein Cholesterol; LDL-C, Low Density Lipoprotein Cholesterol. intracerebral hemorrhage after thrombolysis.

### Predictive value of model constructed by four ML methods

**Figure 2:**
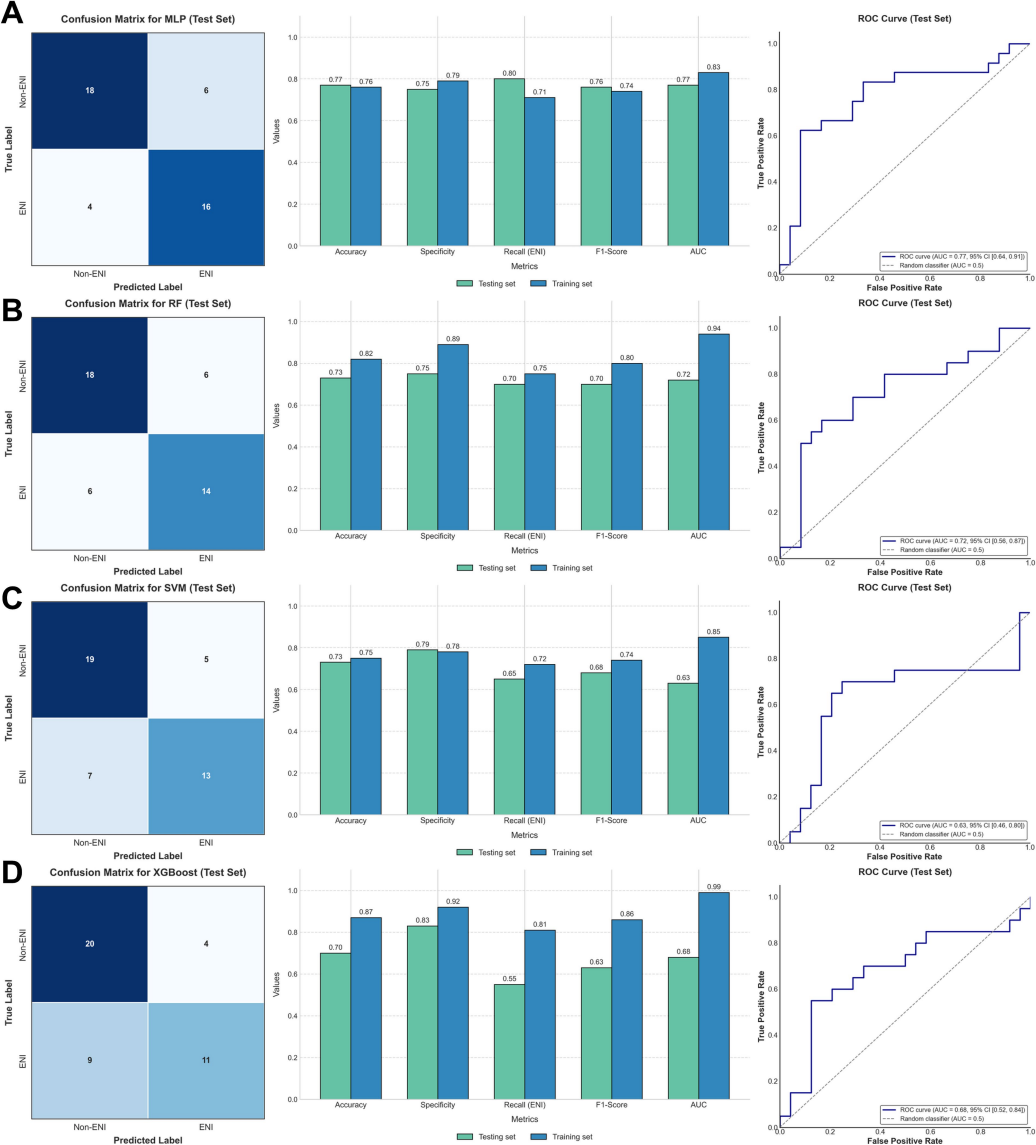
Confusion matrices of the test set (left), comparison of metrics between the training and test sets (middle), and ROC (right) in **(A)** MLP; **(B)** RF; **(C)** SVM; **(D)** XGBoost.

Four ML methods (MLP, RF, SVM, and XGBoost) were used to construct predictive models for ENI using the clinical data. The dataset was divided into training (80%) and testing (20%) sets, comprising 173 and 44 patients, respectively. Using default parameters, all four ML methods demonstrated moderate predictive performance. The AUC values for the training set were 0.83 (MLP), 0.94 (RF), 0.85 (SVM), and 0.99 (XGBoost), while those for the testing set were 0.77 (MLP), 0.72 (RF), 0.63 (SVM), and 0.68 (XGBoost) (Figure 2; Table 2). These results indicate that MLP achieved relatively higher predictive ability compared to the other methods.

**Table 2:** Predictive value of four machine learning models.

Model	Training set (*N* = 173)				Testing set (*N* = 44)			
	MLP	RF	SVM	XGBoost	MLP	RF	SVM	XGBoost
Accuracy	0.76	0.82	0.75	0.87	0.77	0.73	0.73	0.70
Specificty	0.79	0.89	0.78	0.92	0.75	0.75	0.79	0.83
Recall	0.71	0.75	0.72	0.81	0.80	0.70	0.65	0.55
F1-Score	0.74	0.80	0.74	0.86	0.76	0.70	0.68	0.63
AUC	0.83	0.94	0.85	0.99	0.77	0.72	0.63	0.68

### Establishment of predictive model based on the parameters from ML models

**Figure 3:**
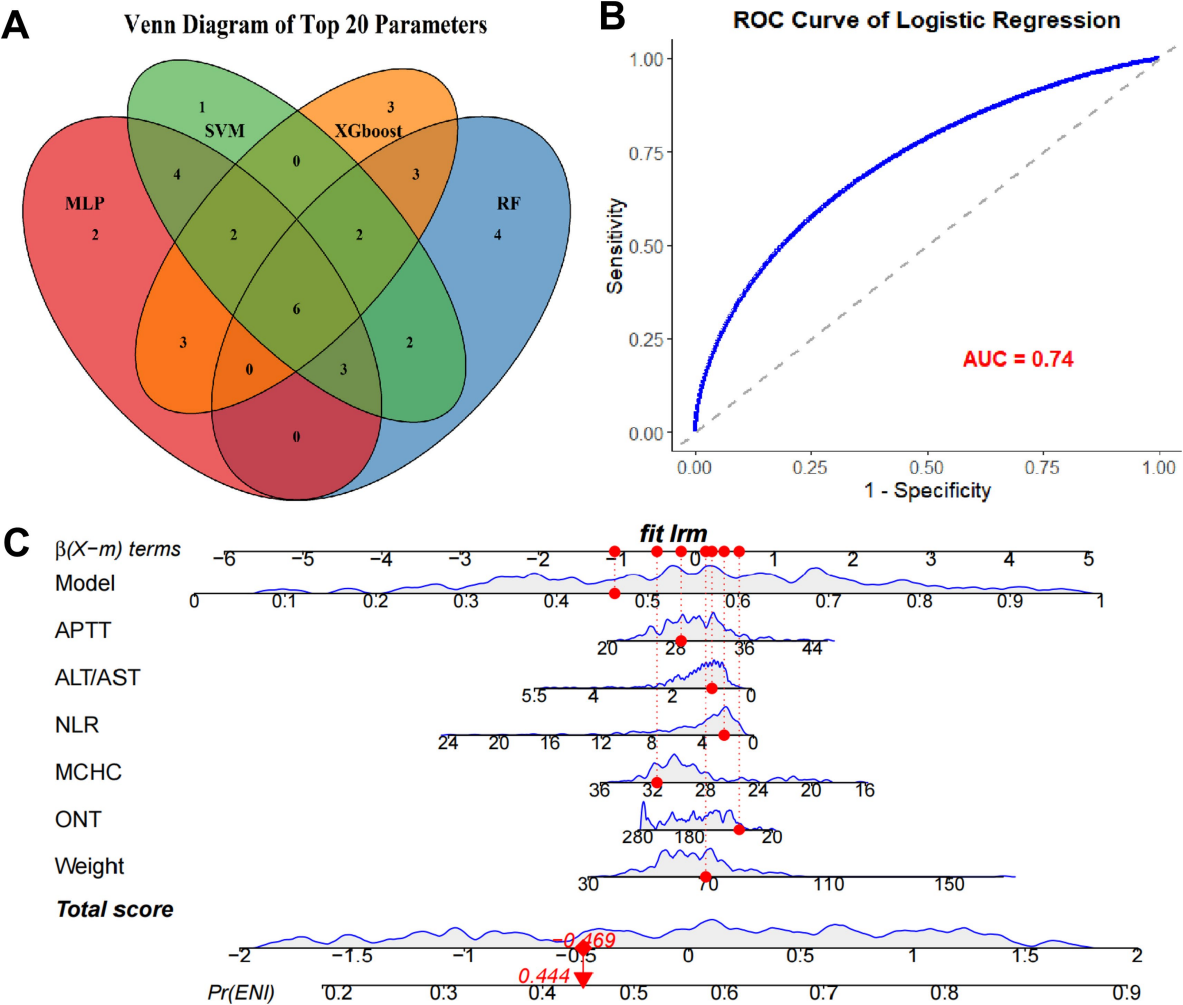
Establishment of predictive model based on the parameters from ML models. **(A)** Venn plot of the overlapping top 20 clinical parameters from each ML model. **(B)** ROC curve of the logistic regression model using the common clinical indexes. **(C)** Nomogram of the predictive model and the six parameters.

To refine the predictive model, we identified the top 20 parameters from each of the four ML models and overlapped them, resulting in six common parameters: APTT, ALT/AST, ONT, MCHC, Weight, and NLR (Figure 3A). The VIF value for each parameter was APTT: 1.32; ALT/AST: 1.45, ONT: 1.28, MCHC: 1.36, Weight: 1.30, and NLR: 1.41. This conservative selection reduced the feature space from near 70–6, yielding an events-per-variable (EPV) ratio of 16.2, which supports model stability. A logistic regression model was then constructed using these six parameters, yielding an AUC value of 0.74 (Figure 3B), which indicates moderate predictive performance. A nomogram was developed to visualize the predictive value of the model relative to the six parameters (Figure 3C), demonstrating that the composite model outperformed individual parameters in predicting ENI, with a bootstrap-corrected C-index of 0.817. these results suggesting that this model show good predictive accuracy for ENI in rt-PA-treated AIS patients. The sensitivity, specificity, PPV, NPV at optimal threshold were listed in Table 3.

**Table 3:** Sensitivity, specificity, PPV, NPV at optimal threshold.

Metric	Value (95% CI)
AUC	0.77 (0.68–0.86)
Sensitivity	72.4% (58.3–83.4%)
Specificity	70.1% (58.9–79.6%)
PPV	58.3%
NPV	81.2%
Calibration slope	0.98
Hosmer-lemeshow p	0.42

### SHAP analysis for the model

**Figure 4:**
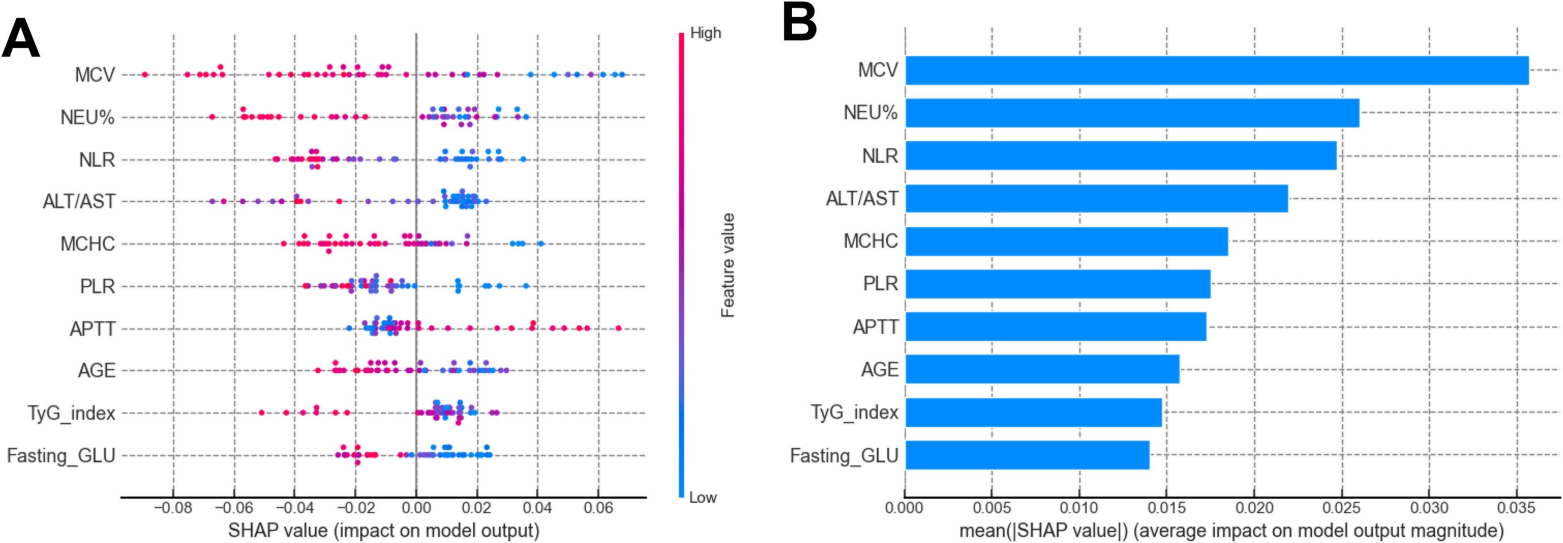
**(A)** SHAP summary plot of the top 10 features of the RF model. The higher the SHAP value of a feature (x-axis), the higher the probability of ENI in AIS patients undergoing rt-PA treatment. Feature values are represented in color (red for high, blue for low). **(B)** SHAP bar plot of the top 10 features of the RF model. The x-axis shows the mean absolute SHAP value, representing the average impact of each feature on the probability of ENI. Features are ranked by importance.

The SHAP analysis elucidated the direction and relative importance of predictive factors influencing ENI after thrombolysis, Figure 4 listed the SHAP summary plot of the top 10 features of the RF model. Among all models evaluated, APTT emerged as the most influential positive predictor, where higher values were consistently associated with better ENI outcomes, suggesting that moderately prolonged coagulation may facilitate neurorecovery post-thrombolysis. In contrast, ALT/AST ratio and MCHC demonstrated significant negative impacts across multiple models (MLP, RF, SVM, XGBoost), implying that liver dysfunction and increased blood viscosity may hinder neurological recovery. Additionally, NEU% and age was consistently associated with poorer ENI outcomes in RF, SVM, and XGBoost models, highlighting the detrimental effects of systemic inflammation and advanced age on prognosis. Notably, RBC and body weight showed positive associations in certain models (e.g., MLP, SVM), possibly reflecting beneficial hemodynamic effects. Conversely, ONT and fasting GLU levels were linked to unfavorable outcomes, underscoring the importance of timely intervention and metabolic control. In summary, APTT, ALT/AST, MCHC, NEU%, and age were identified as the most influential predictors, with their directional effects providing critical insights for risk stratification and personalized therapeutic strategies in thrombolysis management.

## Discussion

Currently, limited evidence is available for the prediction of ENI in AIS patients undergoing rt-PA treatment. The present study conducted a comprehensive analysis by a larger sample of patients to identify significant differences in various clinical and biochemical parameters between ENI and non-ENI groups. The ENI group, consisting of 97 patients, exhibited lower levels of Hemorrhage, ONT, MCV, NEU, NEU%, NLR, and Fasting GLU, while higher levels of Weight, BMI, LYM, LYM%, LMR, A/G ratio, PA, CHE, and APTT were observed compared to the non-ENI group. These findings suggest that these indices are closely associated with ENI, whereas resting indexes did not significantly differ between the two groups, indicating their limited impact on ENI development.

To further explore the predictive capacity of ML models for ENI, we employed four ML algorithms, including MLP, RF, SSVM, and XGBoost, using the common clinical data. By dividing the patients into an 8:2 training-to-testing ratio, the MLP model demonstrated the highest predictive performance with an AUC of 0.77 in the testing set, outperforming RF (0.72), SVM (0.63), and XGBoost (0.68). Subsequently, by intersecting the critical parameters selected by all four ML methods, we identified six common parameters (APTT, ALT/AST, ONT, MCHC, Weight, and NLR) that were then used to construct a logistic regression model. This refined model achieved an AUC of 0.74, indicating its robustness in predicting ENI. Notably, the nomogram based on these six parameters showed a markedly improved predictive performance compared to individual parameters, underscoring the value of this composite approach.

In the present study, we chose the intersection of top-ranked features across multiple ML algorithms as our primary feature selection strategy for several methodological and clinical reasons. This is because different ML algorithms have distinct biases in feature importance estimation. This consensus approach enhances reproducibility. In addition, methods like LASSO are sensitive to multicollinearity and may arbitrarily select one variable from a correlated group. SHAP values, while interpretable, can be computationally intensive and sensitive to model choice. Our intersection method provides a model-agnostic consensus, reducing dependency on any single algorithm’s output. Finally, we provide the Venn diagram to visually justify the selection, enhancing interpretability for clinicians.

The six predictors in our nomogram exhibit strong pathophysiological plausibility. APTT reflects intrinsic coagulation pathway activity; prolonged APTT may indicate impaired clot lysis or re-occlusion post-thrombolysis, increasing ENI risk. NLR is a well-established marker of systemic inflammation, which exacerbates blood–brain barrier disruption and cerebral edema after ischemic stroke. ONT is a critical determinant of tissue viability; delays beyond 4.5 h are associated with reduced reperfusion success and higher complication rates. ALT/AST ratio may reflect hepatic metabolic capacity and redox state, potentially influencing drug metabolism and oxidative stress. MCHC and weight may serve as proxies for nutritional status and comorbidity burden, which are known to affect stroke outcomes. Notably, we used the ALT/AST ratio rather than the more conventionally reported AST/ALT (De Ritis) ratio. While these ratios are mathematically reciprocal, their interpretability in predictive modeling differs. In our machine learning framework, the ALT/AST ratio demonstrated higher feature importance and better discrimination for ENI compared to the AST/ALT ratio. A lower ALT/AST ratio reflects relatively elevated AST levels, which may indicate subclinical hepatic dysfunction, increased oxidative stress, or systemic inflammation, the conditions known to impair neurovascular recovery after ischemic stroke (18, 19). Emerging evidence suggests that an elevated De Ritis ratio (low ALT/AST) is associated with increased infarct volume, hemorrhagic transformation, and poor functional outcomes in AIS (18, 19). This aligns with our finding that a lower ALT/AST ratio is negatively associated with ENI, reinforcing its role as a biomarker of metabolic vulnerability. Furthermore, ALT is predominantly expressed in hepatocytes, while AST is present in multiple tissues including brain, heart, and skeletal muscle; thus, a shift in this ratio may reflect multi-organ stress responses that modulate post-stroke recovery (20).

At present, the clinical application of this ML-based predictive model is substantial to the clinicians. It enables healthcare providers to identify AIS patients who are more likely to experience ENI after rt-PA treatment, thereby facilitating personalized care plans and timely interventions (21, 22). By leveraging the predictive power of the identified parameters, clinicians can optimize patient selection for thrombolysis, enhance monitoring strategies, and potentially improve outcomes. Moreover, the model’s ability to predict ENI may contribute to reducing the risk of adverse events and improving resource allocation in clinical settings (23, 24). Addition, the nomogram in our study could be used in a clinical setting to aid in decision-making and patient counseling. For example, clinicians can input this patient’s specific variables, such as APTT, ALT/AST, ONT, MCHC, Weight, and NLR, to generate a personalized probability of outcome. Suppose the nomogram-predicted risk is 75%. This high estimated risk may prompt earlier initiation of aggressive therapy or enrollment in a clinical trial, whereas a predicted risk of 20% might support a strategy of active surveillance. In patient counseling, this visual and quantitative tool can help clinicians clearly communicate individual risk, facilitating informed discussions about the potential benefits and harms of different management options. Future studies should focus on validating the model across diverse populations and integrating it into clinical decision support systems to maximize its utility in real-world practice.

Previously, there were studies using ML methods to construct predictive model of stroke outcomes (25, 26), but no study using ML methods to construct predictive model for ENI. In addition, although some previous studies have identified several clinical variables associated with ENI (27, 28), to knowledge, our study was firstly using multiple ML algorithms to construct predictive for ENI in AIS patients undergoing rt-PA treatment. In addition, our results revealed that this model showed a moderate predictive performance, which was more accuracy than previous studies that only present the variables associated with ENI. More importantly, unlike some expensive tests, the clinical indexes used to construct the model are common and cheap in clinical practice, thus it is easy for the clinical doctors to construct the predictive model, and it also did not add the addition burden on the patients. Finally, the nomogram enables the clinician to easy distinguish the patients with high risk of ENI. Therefore, our results hold promise for the precision medicine approaches in AIS patients undergoing rt-PA treatment.

To reduce the risk of overfitting of the model, our study used multiple, complementary strategies throughout the modeling pipeline to mitigate this risk. To reduce optimism in performance estimates, we implemented a strict train-test split (80%: 20%) and reported performance only on the held-out test set. The significant drop in AUC from training (XGBoost: 0.99) to testing (0.68) clearly indicates overfitting in some models, which is why we selected the MLP model (AUC 0.77), the most stable performer across training and test sets, as our primary ML model. In addition, rather than using all the variables in the final model, we drastically reduced dimensionality by selecting only 6 overlapping features from the top 20 of four diverse ML algorithms. This conservative selection was designed precisely to combat overfitting. Third, although we used a final logistic regression model, the intersection-based feature selection acts as a form of implicit regularization by selecting only features consistently ranked high across multiple algorithms, reducing the inclusion of spurious associations. Finally, the nomogram’s C-index (0.817) was bootstrap-corrected, meaning it was adjusted for overfitting using internal validation with 1,000 bootstrap resamples. This provides a more realistic estimate of model performance on new data.

In our study, the VIF value of the six parameters were all less than 2 (range: 1.28–1.45), indicating no significant multicollinearity. We note that although some original laboratory parameters (such as AST, ALT, NEU, LYM) are biologically related, our feature selection strategy prioritized composite indices (including ALT/AST ratio, NLR) over individual components, thereby reducing redundancy and enhancing model stability.

Nevertheless, our study also has many limitations. First, while our model demonstrates acceptable discrimination and calibration in internal validation, the retrospective design, single-center setting, and lack of external validation limit its generalizability. Second, the algorithm was built from the input features, and some hidden relationships may have been ignored because unknown or neglected features were not evaluated by physicians. Third, the patient’s long-term prognosis results were not collected. Fourth, the ML algorithms have its own limitation, which can suffer from overfitting, where models perform well on training data but fail to generalize to new, unseen data. Additionally, these methods often lack transparency, making it difficult to interpret the decision-making process, which can be a significant barrier in clinical applications where explainability is crucial (29, 30). Finally, despite our efforts to minimize overfitting, the relatively small sample size (*n* = 217) and high-dimensional feature space pose a risk of overfitting, a common challenge in clinical ML studies. Our findings require external validation in larger, multicenter cohorts to ensure generalizability. Therefore, future study is warranted to verify our results and address the above issues.

## Conclusion

We developed and internally validated a machine learning-based nomogram that shows promising performance in predicting ENI after thrombolysis. The model, incorporating six clinically accessible variables, may serve as a potential tool to support clinical decision-making. However, Future research should focus on external validation and integration of this model into clinical practice to maximize its utility in clinical settings.

## Data Availability

The original contributions presented in the study are included in the article/supplementary material, further inquiries can be directed to the corresponding authors.
